# Implant-supported fixed and removable prostheses in the fibular mandible

**DOI:** 10.1186/s40729-020-00241-7

**Published:** 2020-08-11

**Authors:** Truc Thi Hoang Nguyen, Mi Young Eo, Hoon Myoung, Myung-Joo Kim, Soung Min Kim

**Affiliations:** 1 Department of Oral and Maxillofacial Surgery, Dental Research Institute, School of Dentistry, Seoul National University, 101 Daehak-ro, Jongno-gu, Seoul, 03080 Korea; 2 Department of Prosthodontics, Dental Research Institute, School of Dentistry, Seoul National University, 101 Daehak-ro, Jongno-gu, Seoul, 03080 Korea

**Keywords:** Fibula free flap (FFF), Dental implant, Mandibular reconstruction, Implant-supported prosthesis, Marginal bone loss (MBL)

## Abstract

**Background:**

To restore the health-related quality of life (HRQoL) of patients who underwent jaw resection and reconstruction surgery, dental rehabilitation is an essential procedure and also one of the most challenging for oral and maxillofacial surgeons. Even though recent studies have reported the possibility and reliability of dental implant rehabilitation with the fibula free flap (FFF), clinical reports of long-term follow-up cases are scarce. We herein reported seven cases of FFF reconstruction and implant rehabilitation. We also discussed implant planning strategy and surgical techniques.

**Methods:**

From 2012 to 2019, seven patients were treated with FFF reconstructive jaw surgery combined with dental implant installation and fabrication of implant-supported prostheses at Seoul National University Dental Hospital, Seoul, Korea. Patient characteristics and FFF treatment results were collected. Records of dental implants were analyzed clinically and radiologically.

**Results:**

Among the seven patients in this report, there were three males and four females, with an average age of 54.4 years. A total of 39 implants were placed in the fibular bone. The mean follow-up period after implant installation was 24 months. Five implants failed and were removed 3 months after installation. The implant success rate was 87.2%. Marginal bone loss at 12 months after loading was 0.23 ± 0.18 mm on the mesial side and 0.25 ± 0.26 mm on the distal side.

**Conclusion:**

With the challenges present in FFF-reconstructed patients, an implant-supported prosthesis is a reliable option for stable and functional oral rehabilitation. The implant-supported prosthesis on the FFF has great results regarding restoration of function (mastication, swallowing, and speaking), appearance, and overall HRQoL. Collaboration between surgeons and prosthodontists is essential for a satisfying outcome.

## Background

Since first described in 1975 by Taylor et al., fibula free flap (FFF) has been considered the gold standard technique for reconstruction of mandibular or maxillary deficiencies [1, 2]. The main advantages of FFF are (1) adequate bone stock and bone length to match the maxillary and mandibular defects; (2) ideal vascular pedicle for vessel anastomosis and sufficient skin flap for reconstruction of intraoral and/or extraoral defects; and (3) adequate wide diameter for dental implantation and implant-supported prostheses [3–5]. The indication for FFF reconstruction varies, including reconstruction of bone and soft tissue defects of the oral cavity due to neoplasm ablative treatment, osteomyelitis resection, post-traumatic defects, and congenital facial deformities.

To restore the health-related quality of life (HRQoL) of patients who underwent jaw resection and reconstruction surgery, dental rehabilitation is an essential procedure and also one of the most challenging for oral and maxillofacial surgeons. In FFF reconstructive cases, reconstruction usually offers favorable bony volume and bone quality, which allows for stable dental implant installation and fabrication of functional and aesthetic implant-supported prostheses [6]. Even though there are still several limitations, such as the disadvantages of graft soft tissue and the effects of resection surgery and radiotherapy, recent studies have reported a high success rate, indicating the possibility and reliability of dental implant rehabilitation in FFF [4, 7].

We report seven cases of FFF reconstruction and implant rehabilitation in patients treated for cancer, osteomyelitis, ameloblastoma, or facial deformity. We also discuss the implant planning strategy and surgical techniques in this study.

## Methods

### Patient selection

This study is reported following the Strengthening the Reporting of Observational Studies in Epidemiology (STROBE) guidelines [8]. Among the patients who visited Seoul National University, Oral and Maxillofacial Surgery Department, those with fibula free flap surgery were identified using the electronic medical record (EMR) and Ordering Communication System (OCS). The study protocol and access to patient records were approved by the Institutional Review Board of Seoul National University, Seoul, Korea.

After the initial screening, records of a total of 57 patients who underwent fibula free flap treatment by a single surgeon (SMK) were obtained. Among those 57 patients, nine patients received the implant installation. Only patients who had full surgical and follow-up records were included. All surgical procedures (reconstructions and implant installations) were performed by one surgical team, and patient records were retrospectively reviewed with the following inclusion criteria:

1. Age > 18
2. Receipt of FFF.
3. Treatment with a two-stage surgical protocol.
4. Fabrication and delivery of prosthesis following implant installation.
5. Clinical and radiogram data were available for all treatment periods and follow-up visits.

After applying the inclusion criteria, a total of seven patients were selected. The main exclusion reasons were lacking data regarding prosthesis fabrication and lacking follow-up record.

**Fig. 1:**
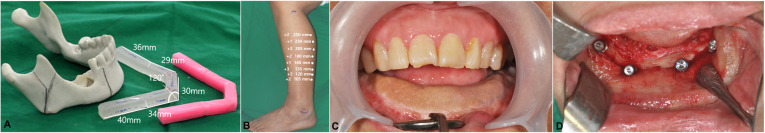
The wax and resin stents were designed based on the RP model of the patient’s mandible and were bent twice at the premolar area. The required bone length was estimated (**a**). Lateral view of patient’s left leg marking the septocutaneous perforators and showing the intensity of the flow determined by a Doppler flowmeter (**b**). Intraoral view 6 months after reconstruction surgery (**c**). Installation of four implants in the fibula bone (**d**)

All the surgical procedures included two stages: reconstruction surgery and implant installation surgery (Fig. 1).

### Reconstruction surgery

**Fig. 2:**
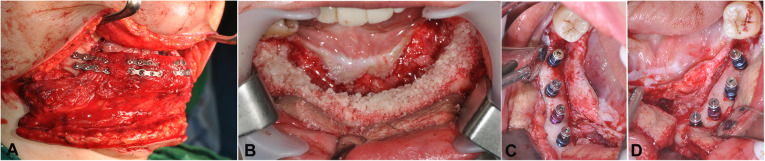
The modeled fibula flap was inserted between the mandibular stumps and fixed with plates and screws (**a**). Bone graft was performed to create adequate bone dimensions for implant insertion (**b**). Eight implants were inserted in the fibula bone (**c**, **d**)

Prior to FFF reconstruction surgery, patient medical history was obtained. Meticulous examinations were performed, including standard clinical examination, orthopantomography, computerized tomographic scans, study models, blood tests, and angiography of the legs. The surgery was performed under general anesthesia. According to disease staging and pre-operative evaluation, the mandible was exposed as necessary for bone resection with a clear margin and for inserting and fixing the reconstruction flap. The lesion was resected with a safe margin, and the FFF was harvested. A plastic template was used to model the flap and helped to decide the number of fragments, as well as their length and orientation. The fibula bone was contoured and modeled according to the shape of the template. The bony fragments were fixed to each other by plate and screws. The modeled fibula flap was inserted between the mandibular stumps and fixed with plates and screws (Fig. 2a). Anastomosis, wound closing, and suturing were performed after fixation of the bone. The donor site was closed with split-thickness skin grafts.

### Implant installation and prosthetic fabrication

If radiotherapy was indicated, implant placement was delayed for 6 to 12 months postoperative radiotherapy (PORT). Due to the specific characteristic of graft tissues, adjunctive surgical procedures were performed to reshape irregular bone or for de-bulking and defatting of the flap. Guided bone regeneration with allograft bone was performed to create sufficient bone volume and bone height if required (Fig. 2b). Soft tissue correction surgery was also performed to obtain adequate vestibular depth and release scars. Plates and screws that interfered with the desired implant position were removed.

Patients underwent implant placement under general or local anesthesia. The implants used were either Straumann® tissue level implant (Straumann® Dental Implant System, Institute Straumann AG, Basel, Switzerland), Luna® self-tapped bone level implant (Shinhung Co., Seoul, Korea), or Stella® self-tapped tissue level implant (Shinhung Co., Seoul, Korea). The number of implants was decided based on prosthesis design and length of edentulous span (Fig. 2c, d). The re-entry surgery was performed 4 to 6 months after installation. After that, once proper conditioning of the soft tissues was obtained, the prostheses were fabricated and delivered. The indicated prosthesis types were conventional partial denture, bar-retained or O-ring-retained overdenture, and screw-retained or cement-retained fixed prosthesis.

### Criteria of success and survival

The criteria of success and survival were based on the health scale for dental implants from The International Congress of Oral Implantologists (ICOI) Pisa Consensus Conference (2008) [9]. The success criteria of an implant were (1) no pain or tenderness upon function, (2) no mobility, (3) < 2 mm radiographic bone loss from the initial surgery, and (4) no exudate history. An implant was considered “failure” if there was one of the following clinical conditions: (1) implant-related pain on function, (2) mobility, (3) radiographic bone loss > 1/2 length of the implant, (4) uncontrolled exudate, and (5) the implant is no longer in the mouth [9]. The survival implants were all the implants that were not considered as “failure” (including success with optimum health and compromised survival implants).

### Marginal bone loss (MBL) measurement

Marginal bone loss (MBL) was measured on panoramic radiogram using image analysis software (Image J®, National Institutes of Health, USA). The marginal bone level was determined as the distance from the implant platform to the first contact point of bone with the implant surface. MBL was determined as the difference between the marginal bone level at a measured time-point and the marginal bone level at installation. The MBL was measured on follow-up radiographs at 6 months and 12 months after loading.

### Pre- and post-operative functional evaluation

The pre-operative functional evaluation was performed to evaluate the residual function of the patient. If there were limitations in mouth opening or lip seal maintenance, patients would be instructed to practice before receiving any dental rehabilitation. The functional evaluation after loading of the prosthesis was also performed to access the chewing, swallowing, speech, and saliva control. The postoperative aesthetic was also evaluated. Based on the evaluation method of Raoul et al. [10], we scored the patient conditions as “excellent” = 2, “good” = 1, and “bad” = 0. The overall total and percentage scores were calculated.

### Statistical analysis

Both descriptive and quantitative data were collected. Means and standard deviations (SDs) of MBL were calculated. The differences between MBL in follow-up time-points were tested by paired Student’s *t* test**.** All analyses were carried out using SPSS (SPSS 25.0®; SPSS Software Company, Chicago, IL, USA). *P* values < 0.05 were considered statistically significant.

## Results

### Reconstruction and implant data

**Fig. 3:**
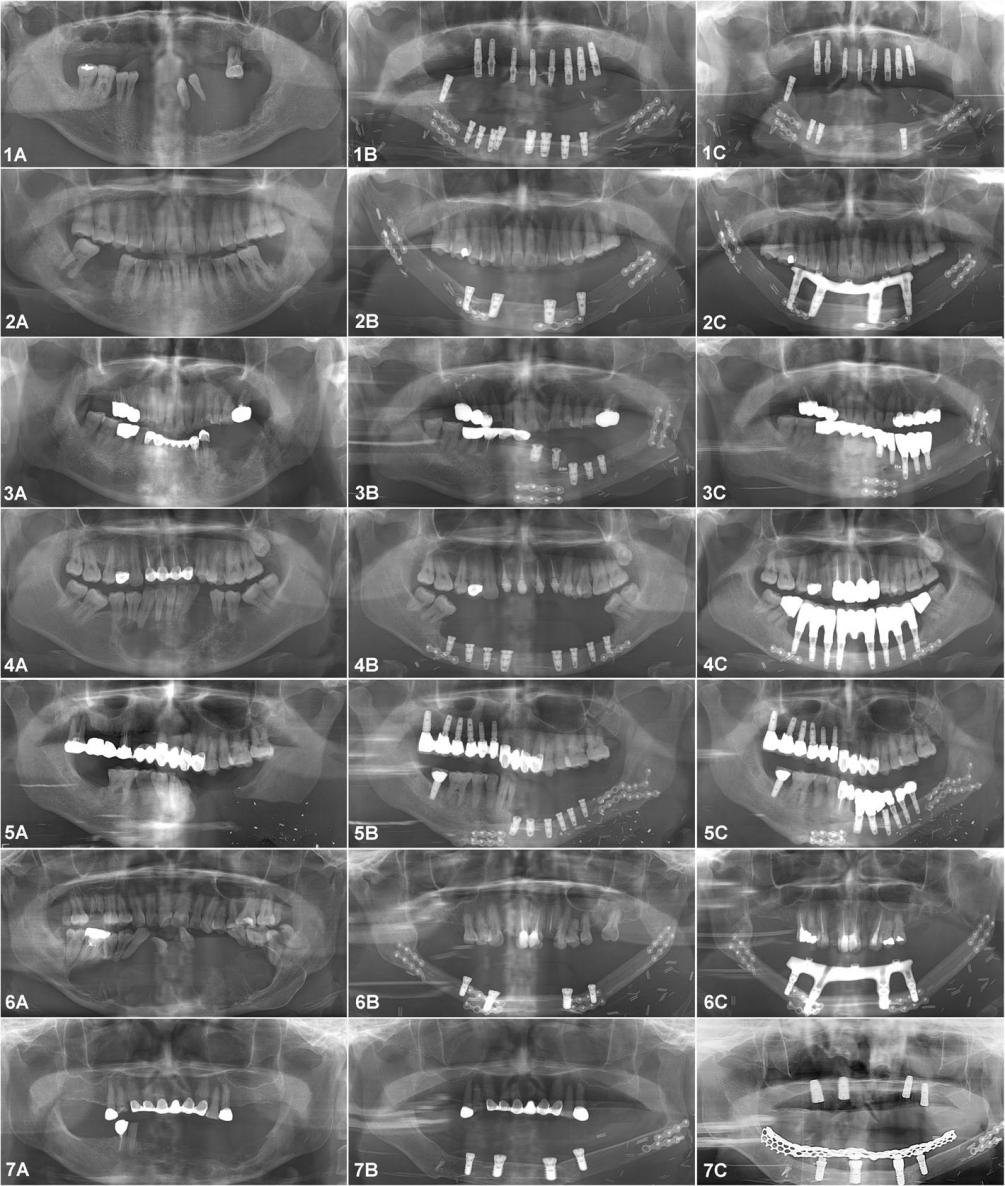
Panoramic view of the seven patients treated with FFF reconstruction and implant rehabilitation. Pre-operative view (**a**). After implant installation (**b**). Follow-up view (**c**)

Among the seven patients in this report, there were three males and four females, with an average age of 54.4 years. All of the patients in this study had a defect in the mandible. None of the patients underwent condylectomy. Two patients had osteomyelitis, two had ameloblastoma, two had cancer, and one had the hemi-face defect due to a gunshot (Fig. 3). All patients were treated with a mono-barrel fibula free flap for mandibular reconstruction. There were no recorded flap failures or complications. No patient was reported to have a recurrence of pathology. The average follow-up period of the fibula free flap was 64.3 months (Table 1). Only one patient received post-operative radiotherapy.

**Table 1:** Patient and reconstruction data

Patient no.	MH	Age/sex	Diagnosis	Treatment	Jaw	Defect region	PORT	GBR with allograft bone	FFF follow-up
01	HTN, ICA stenosis	52M	SCC	Mass resection with subtotal glossectomy and partial mandibulectomy, Lt. RND, Rt SOHND Reconstruction with RFFF (Lt.), FTSG, STSG Saphenous vein graft, tracheostomy, gastrostomy Reconstruction with FFF	Mandible	From #46 region to the Lt. mandible angle	Yes	No	52 months
02	–	47F	OM, BRONJ	Mandibulectomy, reconstruction with FFF (Lt.)	Mandible	From middle of the Rt. ramus to Lt. mandibular angle	No	Yes	46 months
03	2003: adenocarcinoma (Lt. SMG)	78M	Chronic OM	Partial mandibulectomy, reconstruction with FFF	Mandible	Lt. parasymphysis to Lt. condyle	No	Yes	54 months
04	–	35F	Ameloblastoma	Mass resection, partial mandibulectomy, reconstruction with FFF	Mandible	Body of the mandible (35–45)	No	Yes	56 months
05	–	55M	Hemiface defect with facial asymmetry due to gunshot	Mandibular trimming with partial mandibulectomy Reconstruction with FFF, STSG	Mandible	From #42 to Lt. mandibular angle	No	No	88 months
06	Mental retarded	39F	Ameloblastoma	Mass resection, partial mandibulectomy, reconstruction with FFF, STSG	Mandible	From Rt. mandibular angle to Lt. mandibular angle	No	Yes	71 months
07	HTN	75F	Clear cell odontogenic carcinoma	Partial mandibulectomy, SND, recon with FFF, tracheostomy	Mandible	From #46 region to the Lt. mandible angle	No	Yes	69 months

*MH* medical history, *PORT* post-operative radiotherapy, *GBR* guided bone regeneration, *HTN* hypertension, *ICA* internal carotid artery, *SCC* squamous cell carcinoma, *RND* radical neck dissection, *SOHND* supraomohyoid neck dissection, *SND* selective neck dissection, *FTSG* full-thickness skin graft, *STSG*, split-thickness skin graft, *FFF* fibula free flap, *BRONJ* bisphosphonate-related osteonecrosis of the jaw, *SMG* submandibular salivary gland *OM* osteomyelitis

A total of 39 implants were inserted in the fibular bone (Table 2). Of these, there were 27 bone level implants (Luna®) and 12 tissue level implants (Stella® or Straumann®). All the implants achieved initial stability after installation. After insertion, all implants were submerged. The mean follow-up period after implant installation was 24 months. The mean follow-up period after loading was 12.3 months. Five implants were found mobile in patient no. 01 and were removed 3 months after installation. The implant survival rate was 87.2%. There was also no recorded pain or tenderness upon function, no peri-implantitis condition, and the MBL of all survival implants was in 2-plus mm. Therefore, in our study, all the survival implants were generally considered as success implants.

**Table 2:** Inserted implant data

Patient no.	Number of implants	Implant size (mm)	Failed implant	Prosthesis	Implant follow-up	Loaded implant follow-up
01	8	Luna 4.0 × 7.0 (2ea) Luna 4.0 × 8.5 (6ea)	5	Conventional partial denture	32 months	14 months
02	4	Stella 4.0 × 8.5 (3ea) Stella 4.5 × 8.5 (1ea)	None	Bar-retained overdenture	24 months	12 months
03	5	Luna 3.5 × 8.5 (1ea) Luna 4.0 × 7.0 (3ea) Luna 4.0 × 8.5 (1ea)	None	Bridge	28 months	14 months
04	8	Luna 3.5 × 8.5 (2ea) Luna 4.0 × 7.0 (4ea) Luna 4.0 × 8.5 (2ea)	None	Bridge	26 months	16 months
05	6	Luna 4.0 × 7.0 (1ea) Luna 4.0 × 8.5 (5ea)	None	Bridge (1 implant was buried)	38 months	14 months
06	4	Straumann 4.1 × 8.0 (4ea)	None	Bar-retained overdenture	24 months	12 months
07	4	Straumann 4.1 × 8.0 (4ea)	None	O-ring-retained overdenture	27 months	23 months

Among the seven patients that underwent prosthesis fabrication, four had the conventional denture or overdenture prosthesis and three had the fixed bridge prosthesis. In patient no. 05, one implant did not carry the prosthesis and was left unloaded. Six patients excluding patient no. 01 reported satisfying functional and aesthetic outcomes of the implant-supported prosthesis, but no. 01 patient understood his high PORT effects finally.

### Marginal bone loss (MBL)

MBL values at 6 months after loading were 0.15 ± 0.12 mm on the mesial side and 0.17 ± 0.10 mm on the distal side. MBL values at 12 months after loading were 0.23 ± 0.18 mm on the mesial side and 0.25 ± 0.26 mm on the distal side (Table 3). MBL after 6 and 12 months of loading did not differ significantly (*p* > 0.05).

**Table 3:** Marginal bone loss (MBL) at 6 and 12 months after loading compared to bone level at installation

	MBL at 6 months after loading (mm)	MBL at 12 months after loading (mm)	
Mesial	0.15 ± 0.12	0.23 ± 0.18	*p* = 0.11
Distal	0.17 ± 0.10	0.25 ± 0.26	*p* = 0.12

MBL at 6 and 12 months after loading did not differ significantly (*p* > 0.05)

### Pre- and post-operative functional evaluation

After the mean follow-up period of 12.3 months after prosthesis loading, chewing and swallowing functions showed a significant improvement (percentage score increased from 64.3 to 85.7%) along with the great improvement of occlusion after dental rehabilitation (percentage score increased from 35.7 to 92.9%) (Table 4). The speech also achieved a higher score (percentage score increased from 78.6 to 85.7%). The speech score increased significantly in mandibulectomy patients when the dentures were supporting the lower lip and helped to improve the pronunciation. The aesthetic result of all patients was also scored as “good” to “excellent” with the restoration of the facial outline and profile. Besides, the aesthetic of the lip line was restored.

**Table 4:** Pre- and post-operative functional evaluation

Patient no.	Pre-operative evaluation			Post-operative evaluation			
	Chewing and swallowing	Speech	Occlusion	Chewing and swallowing	Speech	Occlusion	Aesthetic
01	0	1	0	1	1	1	0
02	2	2	2	2	2	2	1
03	2	2	1	2	2	2	2
04	2	2	2	2	2	2	2
05	1	1	0	2	2	2	2
06	1	1	0	1	1	2	1
07	1	2	0	2	2	2	2
Total score	9/14	11/14	5/14	12/14	12/14	13/14	10/14
Percentage	64.3%	78.6%	35.7%	85.7%	85.7%	92.9%	71.4%

Excellent = 2, good = 1, bad = 0

## Discussion

Through a cadaveric study, Klesper et al. [11] reported that the fibula flap is the most suitable flap for reconstruction of mandibular defects, especially a long-span defect, and provides adequate width and bone volume for installation of osseointegrated dental implants. A recent retrospective study of Burgess et al. [12] reported that the success rate of implants placed in fibula flaps was 92% over an average follow-up of 30 months. Sozzi et al. [4] reported a survival rate of 98% with a mean follow-up after implant loading of 7.8 years. Wu et al. [13] reported 1-year and 5-year cumulative survival rates of implants to be 96 and 91%, respectively. The authors determined that the main reasons for implant failure were recurrence of tumor, soft tissue proliferation, and infection. In our current study, the success rate was 87.2% with an average follow-up after surgery of 24 months.

In patient no. 01, five implants were removed 3 months after installation. The main factors that caused the failure of these five implants were PORT effects and the complicated treatment that the patient underwent, which included many microvascular flaps, tracheostomy, and gastrostomy. In addition, it was an aggressive treatment plan involving a large number of implants. Radiation therapy was originally considered a contraindication for the installation of dental implants [14]. Patients treated with radiation therapy present with hypovascular, hypocellular, and hypoxic, as well as decreased saliva, and an increased risk for osteoradionecrosis. Therefore, irradiation has a large impact on the prognosis of patients treated with dental implants. However, many recent studies reported evidence that implants inserted in the irradiated jaw can have a comparable success rate with implants inserted in the non-irradiated jaw with the proper and meticulous management [15, 16]. Other authors also agreed that radiotherapy had a few influences on the success rate of dental rehabilitation in irradiated fibular grafted bone [10, 17]. In the cases that the irradiated dose is more than 50 Gy, implant installation is not recommended due to the high risk of osteoradionecrosis. In patient no. 01, after the removal of five implants, the remaining three implants were in stable status without any complications. After consulting with the patient and considering of bone healing process, the implants were kept submerged and a plan of re-installation with a smaller number of implants was made. A provisional prosthesis was fabricated to restore the function and aesthetic partially.

**Fig. 4:**
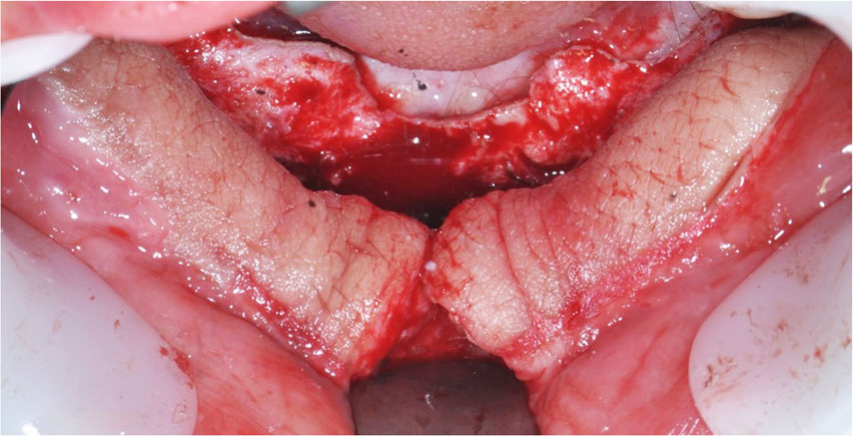
A full thickness flap was elevated, and the skin tissue covering the fibular bone was “defatted”

In our study, not all patients had sufficient bone dimensions for implant insertion after FFF reconstruction surgery. In these patients, guided bone regeneration with allograft bone was performed to create sufficient bone volume and bone height (Fig. 2b). Other adjunctive surgical procedures, such as reshaping surgery and scar releasing, could have been performed if necessary. Reconstructed skin is not an ideal peri-implant tissue [7]. The tissue covering the fibular bone was skin, therefore “defatting” and peri-implant soft tissue management, should be performed carefully (Fig. 4) [18].

In a randomized clinical trial of implants in FFF, Kumar et al. [18] reported MBL of a two-implant-supported-overdenture group to be 0.4 and 0.5 mm at 6 and 12 months, respectively. On the other hand, MBL of a four-implant-supported-overdenture group was 0.1 and 0.2 mm at 6 and 12 months, respectively. The MBL in the current study was 0.15 ± 0.12 mm on the mesial side and 0.17 ± 0.10 mm on the distal side at 6 months after loading. MBL values at 12 months after loading were 0.23 ± 0.18 mm on the mesial side and 0.25 ± 0.26 mm on the distal side. These MBL values are comparable to those of previous studies and to the established success criteria of implants inserted on native mandibular bone.

**Fig. 5:**
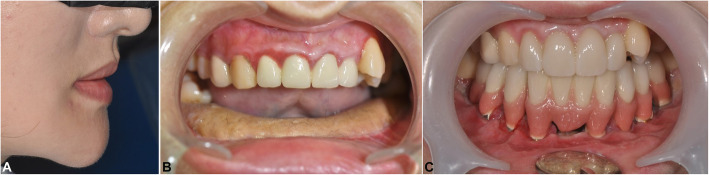
Clinical views of patient no. 04 showed restoration of occlusion and facial profile. Extraoral view after delivery of the prosthesis (**a**). Intraoral view after reconstruction surgery (**b**). Intraoral view after delivery of the prosthesis (**c**)

**Fig. 6:**
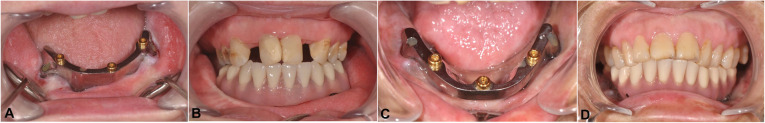
Implant-supported overdentures in the fibular bone graft showed satisfying functional and aesthetic results. Bar-retained overdenture of patient no. 06 (**a**, **b**). Bar-retained overdenture of patient no. 02 (**c**, **d**)

The prosthesis indication of each patient was based on individual conditions, edentulous span, and occlusion. In patient no. 04, eight implants were inserted in a long reconstructed fibula bone. The final implant-supported bridge satisfactorily restored the function (including mastication, swallowing, and speaking) and the patient’s facial profile, despite the large defect after resection of large ameloblastoma (Fig. 5). In the other two patients, bar-retained overdentures were indicated and also presented satisfying functional and aesthetic results (Fig. 6).

**Fig. 7:**
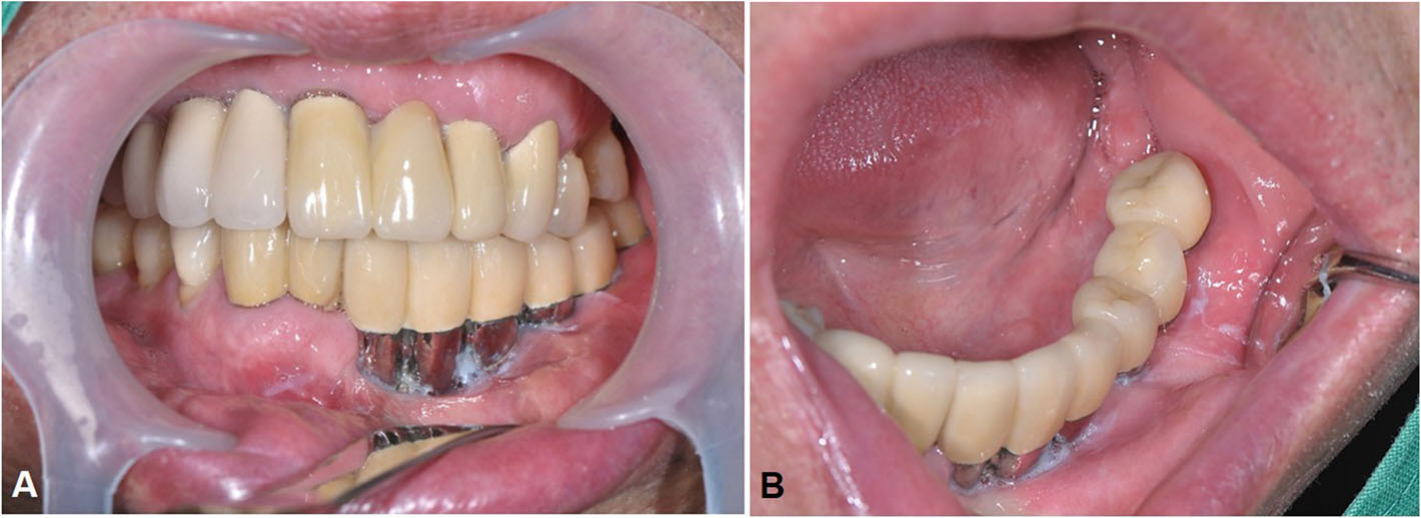
Oral hygiene was checked during follow-up visits. Re-education and shorter waiting time between follow-up periods were indicated for poor oral hygiene patients

It is well established that maintenance of healthy peri-implant soft tissue is essential for preservation of marginal bone stability, and this is even more critical in implant-inserted reconstructed bone [19]. Some characteristics of the extraoral skin flap also cause poor hygiene around implants, resulting in a high risk of chronic inflammation followed by peri-implantitis and MBL (Fig. 7). Kumar et al. [18] observed that hyperplastic peri-implant tissues are common in the early implant-loading phase and tend to decrease over time under appropriate management. The grafted overlying skin and soft tissue tend to have excessive mobility, which can cause an inflammation reaction and require prosthesis margin adaptation.

Patient self-hygiene of the prosthesis and peri-implant soft tissue is essential for long-term maintenance of dental implants. According to type of prosthesis, patients were introduced to the specific methods of oral hygiene. The patient’s oral hygiene was checked thoroughly during the follow-up visit. If the patient had poor oral hygiene, re-education and shorter follow-up periods were required.

## Conclusion

With the challenges present in FFF-reconstructed patients, an implant-supported prosthesis is a reliable option for stable and functional oral rehabilitation. The implant-supported prosthesis on the fibula free flap has great results regarding restoration of patient function (mastication, swallowing, and speaking), appearance, and overall HRQoL. Collaboration between surgeons and prosthodontists is essential for a satisfying outcome.

## Data Availability

The datasets used and/or analyzed during the current study are available from the corresponding author on reasonable request.
